# Supplementary material for: A mouse model of autosomal dominant spastic ataxia and myopathy caused by a mutation in Tuba4a
Source: bioRxiv. 2026 Mar 9:2026.03.06.710113. Preprint. [Version 1] doi: 10.64898/2026.03.06.710113 (PMC13014175; doi:10.64898/2026.03.06.710113)
Supplement: Supplement 4 [file NIHPP2026.03.06.710113v1-supplement-4.pdf]

bioRxiv preprint doi: https://doi.org/10.64898/2026.03.06.710113; this version posted March 9, 2026. The copyright holder for this preprint (which was not certified by peer review) is the author/funder, who has granted bioRxiv a license to display the preprint in perpetuity. It is made available under aCC-BY 4.0 International license.

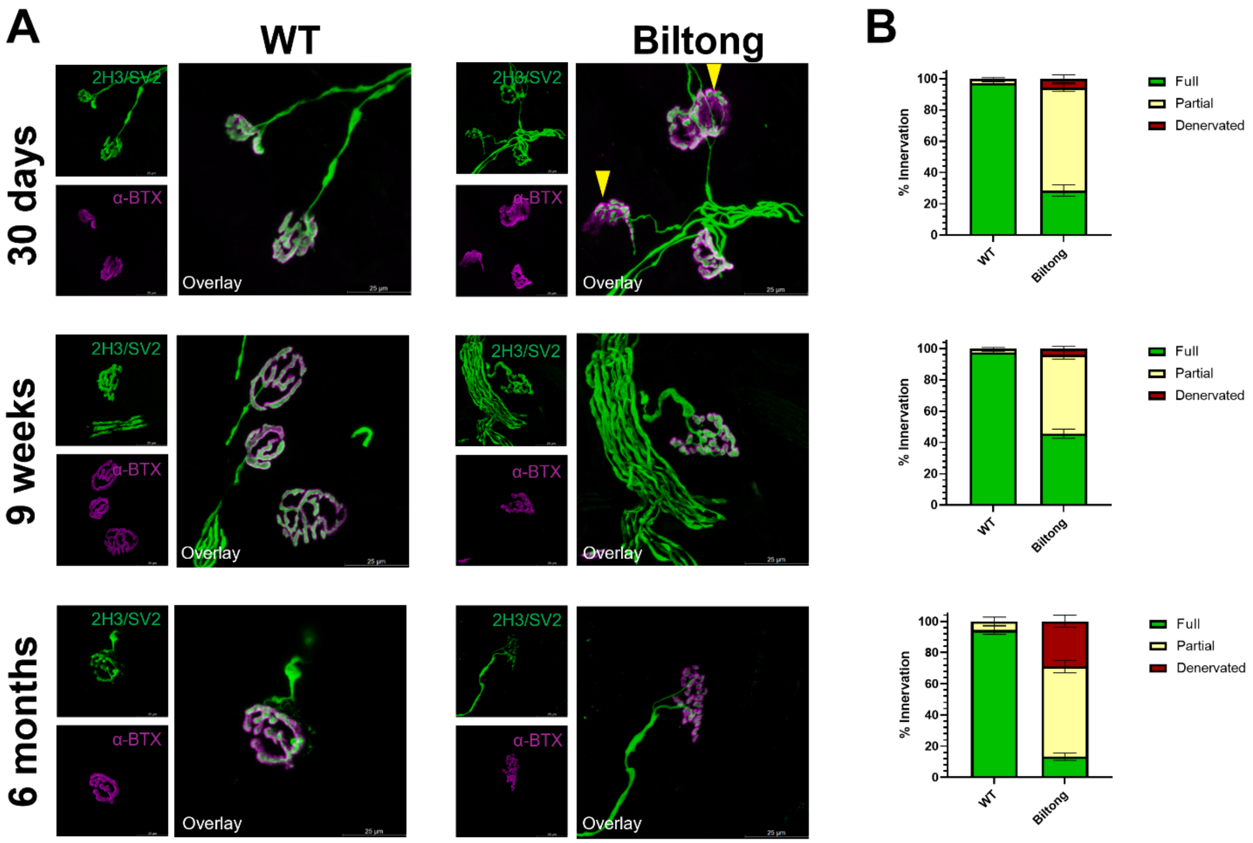

Supplementary Figure 1. NMJ denervation in plantaris of Biltong mice. **A)** Images of plantaris NMJs from WT and Biltong mice at three timepoints (30d, 9wk, and 6mo). The axon terminal/presynapse is labeled in green with a cocktail of SV2 and NFM antibodies, while the acetylcholine receptors/postsynapse are labeled in magenta with AlexaFluor594-conjugated α-bungarotoxin. Yellow arrowheads in 30 d Biltong image indicate partially innervated NMJs. Scale bars are 25 µm. **B)** Quantification of fully innervated (green), partially innervated (yellow), and denervated (red) NMJs from the same mice as in (A). N = 3 WT, 7 Biltong at 30 d; 10 WT, 11 Biltong at 9 wk; and 10 WT, 13 Biltong at 6 mo.

bioRxiv preprint doi: https://doi.org/10.64898/2026.03.06.710113; this version posted March 9, 2026. The copyright holder for this preprint (which was not certified by peer review) is the author/funder, who has granted bioRxiv a license to display the preprint in perpetuity. It is made available under aCC-BY 4.0 International license.

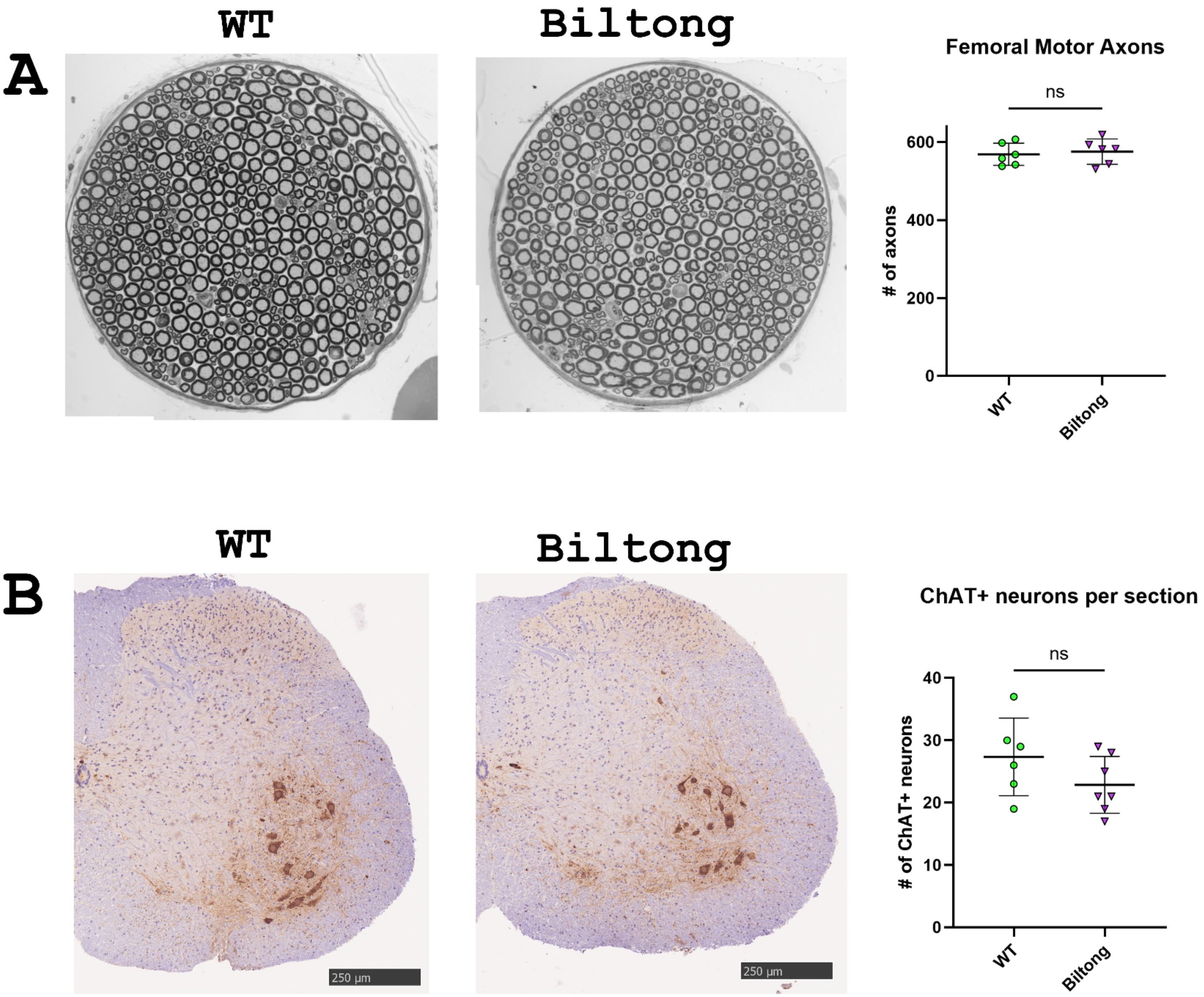

Supplementary Figure 2. Biltong mice do not show signs of motor neuron degeneration at 6 months. **A)** Images of semithin toluidine blue-stained cross-sections of the motor branch of the femoral nerve and quantification of the number of axons in nerves from WT and Biltong mice at 6 months. N = 6 WT and 6 Biltong mice. **B)** Images of ChAT-labeled spinal cord cross-sections and quantification of motor neurons in spinal cord sections from WT and Biltong mice at 6 months. N = 6 WT and 7 Biltong mice.

bioRxiv preprint doi: https://doi.org/10.64898/2026.03.06.710113; this version posted March 9, 2026. The copyright holder for this preprint (which was not certified by peer review) is the author/funder, who has granted bioRxiv a license to display the preprint in perpetuity. It is made available under aCC-BY 4.0 International license.

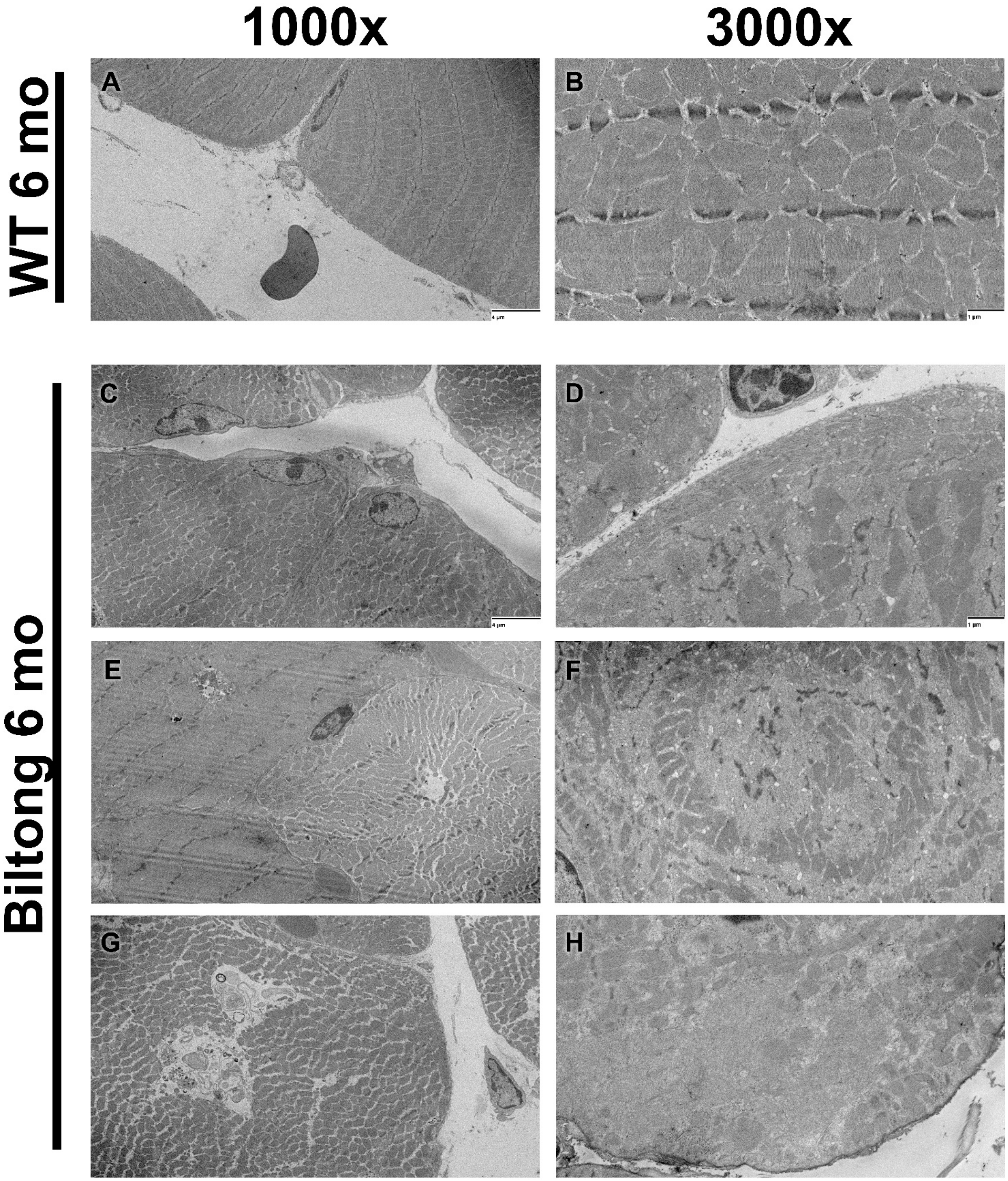

Supplementary Figure 3. No true ringbinden seen with TEM on tibialis posterior from Biltong mice. **A-B)** Representative TEM images of tibialis posterior muscle from WT mice showing normal ultrastructure. **C-H)** TEM images of tibialis posterior muscle from Biltong mice revealed pathology similar to the soleus (Figure 6) including Z-band streaming, osmiophilic inclusions and multilamellar myeloid bodies, and numerous fibers with disorganized myofibrils. However, no fibers were seen with the typical perpendicular re-organization of myofibrils seen in true ringbinden observed in myotonic or limb-girdle muscular dystrophy patients.

bioRxiv preprint doi: https://doi.org/10.64898/2026.03.06.710113; this version posted March 9, 2026. The copyright holder for this preprint (which was not certified by peer review) is the author/funder, who has granted bioRxiv a license to display the preprint in perpetuity. It is made available under aCC-BY 4.0 International license.

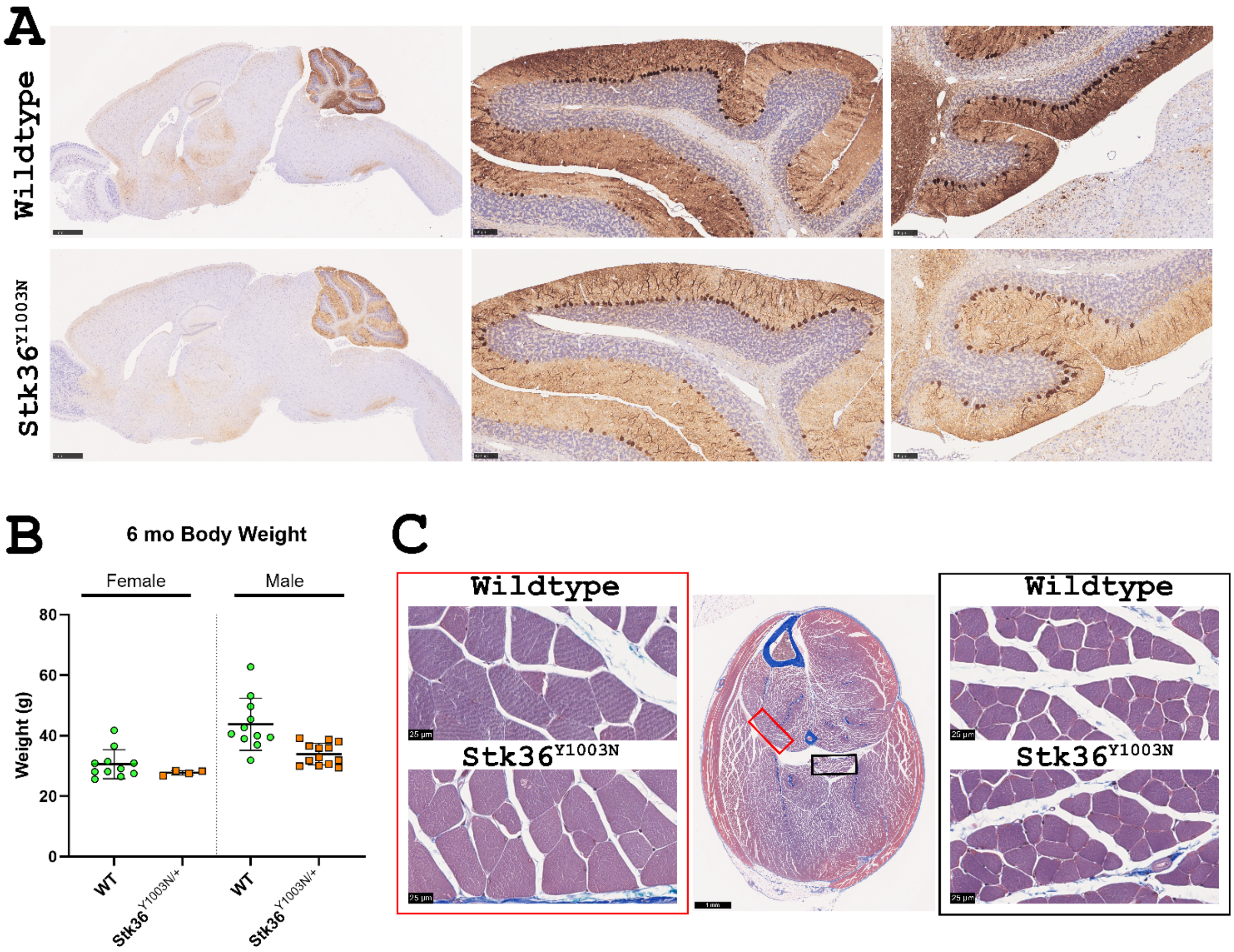

Supplementary Figure 4. *Stk36*^{Y1003N} knockin mouse does not produce a discernible phenotype by 6 months. **A)** Sagittal brain sections from 6 mo WT and *Stk36*^{Y1003N} knockin mice stained with Calbindin to label cerebellar Purkinje neurons. High magnification images show lobules VI (left) and X (right). Scale bars are 1 mm (whole brain) and 100 µm (lobules VI and X). **B)** Body weights from 6 mo WT and *Stk36*^{Y1003N} female and male mice. **C)** Masson's trichrome staining of lower hindlimb cross-sections taken from WT and *Stk36*^{Y1003N} knockin mice at 6 months. High magnification (80x) insets are shown from the tibialis posterior (left, red rectangle) and soleus (right, black rectangle). Scale bars are 1 mm (whole hindlimb section) or 25 µm (80x images).
